# Pediatric Acute Disseminated Encephalomyelitis Triggered by Concurrent Administration of Seasonal and H1N1 Influenza Vaccines: A Case Report and Review

**DOI:** 10.3390/neurosci6010001

**Published:** 2024-12-30

**Authors:** George Imataka, Hideaki Shiraishi, Shigemi Yoshihara

**Affiliations:** Department of Pediatrics, Dokkyo Medical University, Tochigi 321-0293, Japan

**Keywords:** ADEM, MRI, child, influenza vaccine, H1N1, immunological response, autoimmune encephalitis

## Abstract

Background: Acute disseminated encephalomyelitis (ADEM) is a rare, immune-mediated inflammatory disorder of the central nervous system (CNS), typically characterized by the acute onset of multifocal demyelination. The pathogenesis of ADEM remains unclear, but it is believed to be triggered by an autoimmune response, often following viral infections or vaccinations. Case report: This case report describes a 3-year-old child who developed ADEM after receiving two concurrent influenza vaccines: one for seasonal influenza and one for the 2009 H1N1 pandemic. The patient presented with motor regression, mild pleocytosis in cerebrospinal fluid (CSF), and typical MRI findings of ADEM. Steroid pulse therapy resulted in rapid improvement, and the patient recovered fully without sequelae. Results: Although the influenza vaccine has been linked to ADEM in some studies, it remains uncertain whether the simultaneous administration of both vaccines contributed to the onset of ADEM. While influenza vaccines are considered safe and effective by health organizations such as the CDC, data suggest that the incidence of ADEM and other neurological complications is significantly higher after natural influenza infections compared to vaccination. This highlights the importance of vaccination in preventing severe outcomes. Conclusions: This case underscores the importance of monitoring and reporting adverse events following vaccination to refine our understanding of rare complications like ADEM. While simultaneous vaccine administration warrants further research, the benefits of vaccination in preventing severe complications from natural infections far outweigh the risks. Continued vigilance and improved surveillance systems are essential for maintaining public confidence in vaccination programs.

## 1. Introduction

Acute disseminated encephalomyelitis (ADEM) is a rare but serious autoimmune condition that typically follows viral infections or vaccination, manifesting as an acute, monophasic demyelinating disorder of the central nervous system (CNS) [1]. Several viral agents, including the measles, mumps, rubella, and varicella viruses, as well as the influenza virus, have been identified as potential triggers for ADEM [2]. The pathophysiology of ADEM is thought to involve an autoimmune response, where the body’s immune system attacks the myelin of the CNS, following molecular mimicry between viral antigens and myelin components [3,4]. While most cases of ADEM are idiopathic or triggered by infections, a significant number of cases have been reported after vaccination, including vaccines for influenza, measles, and hepatitis B [5,6,7,8].

In particular, the 2009 H1N1 pandemic raised concerns regarding vaccine-related neurological complications, including ADEM. Multiple studies have reported cases of ADEM following H1N1 vaccination, suggesting an immune-mediated response, possibly exacerbated by the simultaneous administration of seasonal and pandemic vaccines [9,10]. This has led to ongoing discussions regarding the safety of concurrent influenza vaccination. Despite the risk, influenza vaccines are generally considered safe and are strongly recommended for both seasonal influenza and pandemic strains. However, the rare occurrence of neurological complications like ADEM emphasizes the need for careful monitoring and further investigation into the mechanisms involved in vaccine-associated CNS inflammation. Understanding these mechanisms is crucial for informing vaccination guidelines and ensuring that risk factors are adequately considered during vaccine administration, especially in pediatric populations with underlying health conditions.

Although ADEM is an extremely rare complication after vaccination, data do not suggest an increased incidence of post-vaccination ADEM. Instead, ADEM is much more frequently observed following natural infections. Understanding the clinical presentation, diagnostic challenges, and the mechanisms of such events is crucial for public health policies. Furthermore, recent studies have reported post-vaccination ADEM after SARS-CoV-2 vaccines, which should also be considered in the broader context of vaccine safety. Furthermore, research continues to explore the immunological factors that predispose certain individuals to ADEM following vaccination, highlighting the need for personalized approaches to vaccine administration, especially for those with underlying immunological vulnerabilities.

In this case report, we describe a 3-year-old boy who developed ADEM after receiving both seasonal and H1N1 influenza vaccines on the same day. The child’s clinical course and diagnostic findings will be discussed in light of the current literature on ADEM and its association with influenza vaccination.

## 2. Case Report

A previously healthy 3-year-old male was admitted to our hospital with a 3-day history of gait disturbance, which was accompanied by difficulty performing routine motor activities such as turning over and walking. The child had received two influenza vaccinations 15 days prior to admission: the seasonal influenza vaccine (BIKEN) and the H1N1 pandemic influenza vaccine (HP04B), in accordance with the recommended immunization schedule for children of his age group. These vaccinations were administered as part of the standard protocol for influenza prevention, with no known contraindications or adverse reactions prior to this event. The child’s family reported no previous history of neurological symptoms, autoimmune disorders, or any significant medical concerns, indicating that his health history had been uneventful until the onset of these symptoms.

Upon admission, the child was unable to perform basic motor functions, including rolling over and walking, which had progressively worsened over the preceding 3 days. Clinical observation revealed decreased muscle tone and an overall diminished ability to perform voluntary movements. These findings were consistent with a significant impairment in motor control, raising concerns about a central nervous system (CNS) pathology affecting motor function. Blood tests performed on admission showed no significant changes in the white blood cell count or C-reactive protein levels, which suggested the absence of systemic bacterial infection. However, further examination of interleukin 2 receptor (IL-2R) levels revealed a marked elevation to 2504 U/mL. Elevated IL-2R levels are commonly seen in autoimmune conditions, suggesting the presence of an active immune response, possibly related to an inflammatory or demyelinating process affecting the central nervous system.

Cerebrospinal fluid (CSF) analysis was conducted to further evaluate the possibility of an infectious or inflammatory CNS disorder. The CSF examination showed mild pleocytosis, with a total cell count of 16 cells/µL, which, while elevated, is still within the upper limits of normal in many clinical settings. This finding is suggestive of an inflammatory response, although it did not reach the levels typically seen in severe infections such as bacterial meningitis or encephalitis. Importantly, no abnormalities were observed in the glucose or protein levels of the CSF, which typically rise in cases of infection or more severe forms of encephalitis. These findings helped to exclude common viral and bacterial CNS infections and indicated the need to consider other potential etiologies, such as autoimmune-mediated encephalitis or demyelination.

**Figure 1:**
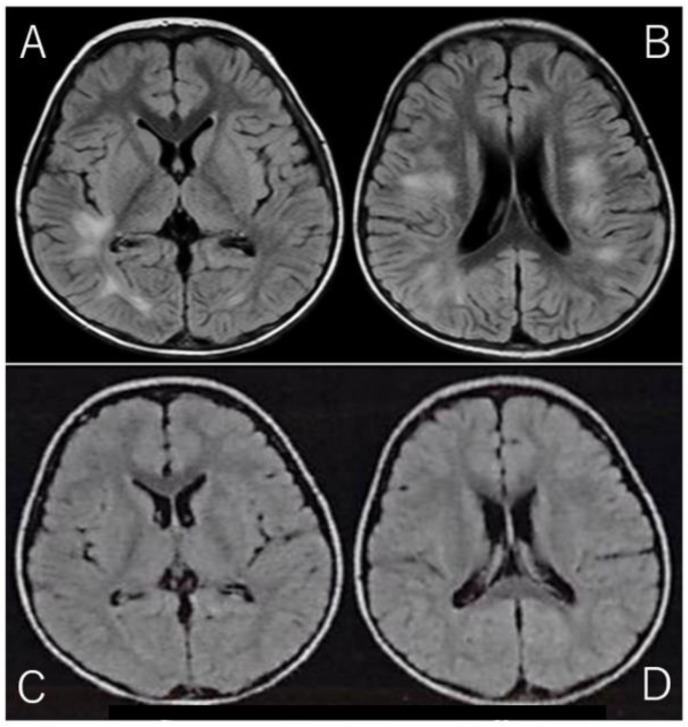
Brain MRI on Admission and Discharge (Spin Echo Method: Fluid Attenuated Inversion Recovery; FLAIR Axial: TR = 9000, TE = 104, FA = 150): (**A**,**B**): On admission, brain MRI with the Fluid Attenuated Inversion Recovery (FLAIR) sequence revealed high-intensity lesions bilaterally beneath the cerebral cortex, with asymmetrical involvement. FLAIR imaging, designed to suppress cerebrospinal fluid signals, provides enhanced visibility of brain parenchyma abnormalities, particularly those involving edema or inflammation. The bilateral, asymmetrical nature of the lesions suggests a possible systemic or multifocal pathology. These lesions are characterized by abnormal fluid accumulation, which appears bright on FLAIR images due to the suppression of CSF, allowing for better delineation of tissue abnormalities. (**C**,**D**): On follow-up MRI at discharge, these lesions had significantly improved, with reduced intensity in the previously affected areas. The reduction in signal suggests a decrease in underlying edema or inflammation, reflecting a positive change in the brain’s condition. This improvement highlights the effectiveness of follow-up imaging in monitoring the resolution of abnormalities over time.

Given the clinical presentation and CSF findings, along with the marked elevation in IL-2R, a diagnosis of acute disseminated encephalomyelitis (ADEM) was suspected. ADEM is a rare but serious autoimmune-mediated demyelinating disorder of the CNS, often triggered by viral infections or vaccinations. The diagnosis was further supported by brain MRI findings, which were obtained using the fluid-attenuated inversion recovery (FLAIR) sequence. FLAIR imaging is particularly useful for detecting abnormalities in the brain’s white matter, as it suppresses cerebrospinal fluid signals and highlights areas of high water content. The MRI revealed high-intensity lesions bilaterally in the periventricular white matter, particularly beneath the cerebral cortex (Figure 1A,B), which is a classic finding in ADEM. These lesions, indicative of acute demyelination, were consistent with the diagnosis of ADEM, a condition that can present with various neurological deficits, including motor dysfunction, altered consciousness, and in some cases, seizures.

Upon confirmation of the diagnosis, the patient was promptly started on a 3-day course of high-dose steroid pulse therapy (30 mg/kg/day), a standard treatment regimen aimed at reducing inflammation and modulating the immune response in cases of ADEM. Corticosteroid therapy is typically the first-line treatment and has been shown to be effective in accelerating recovery and improving functional outcomes in children with this condition. Within 4 days of initiating treatment, the child showed significant clinical improvement. His motor skills began to improve gradually, and he regained the ability to walk independently, which was a considerable relief for both the child and his family. This improvement was consistent with the expected therapeutic response to corticosteroid therapy in ADEM, which typically leads to rapid reduction in inflammation and stabilization of neurological function.

The patient was discharged after a total of 14 days of hospitalization, during which he also underwent physical therapy to address the initial motor difficulties and to support his recovery. Follow-up MRI performed just prior to discharge showed a marked reduction in the high-intensity lesions (Figure 1C,D), which is consistent with the expected resolution of acute demyelination following appropriate treatment. These MRI findings provided further confirmation of the positive treatment response and the expected recovery trajectory in ADEM cases.

At the 2-year outpatient follow-up, the patient remained neurologically intact, with no signs of sequelae such as motor difficulties, cognitive impairment, or recurrent neurological symptoms. The child was developmentally normal and had returned to his usual activities without restrictions. His family reported no concerns, and the child continued to progress without any signs of relapse. Notably, the parents chose not to administer any further influenza vaccinations, citing concerns about a potential recurrence of similar symptoms. While it is recognized that the risk of recurrence after vaccination-related ADEM is low, the family was advised to proceed with caution when considering future vaccinations. They were encouraged to consult with healthcare professionals regarding the potential risks and benefits of further immunizations in light of the child’s previous adverse event.

## 3. Discussion

The association between influenza vaccination and ADEM, although rare, has been documented in several studies. Influenza vaccines, including those for seasonal and pandemic strains, are known to trigger immune responses that could lead to CNS inflammation [9,10,11]. The pathophysiology of vaccine-induced ADEM is thought to involve molecular mimicry, where the immune system mistakenly targets myelin after recognizing structural similarities between viral proteins and myelin components [12,13]. In addition to ADEM, other neurological complications such as Guillain–Barré syndrome and transverse myelitis have been linked to influenza vaccination [14,15,16].

**Figure 2:**
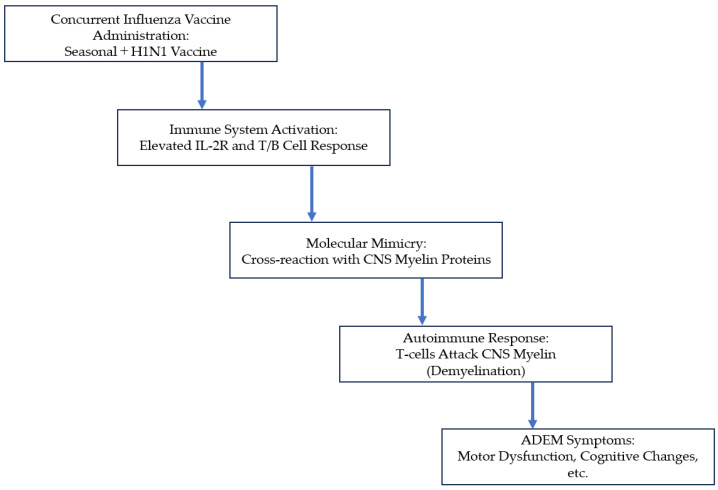
Proposed Mechanism of Vaccine-Induced Acute Disseminated Encephalomyelitis (ADEM).

The risk of developing ADEM after vaccination remains extremely low, but the potential for serious neurological sequelae underscores the importance of monitoring vaccine recipients for adverse events, particularly in vulnerable populations such as young children and individuals with a history of autoimmune disorders [11]. Despite the theoretical concerns regarding immune responses triggered by simultaneous vaccine administration, there is no conclusive evidence from the literature to support a significant increase in the incidence of ADEM post-vaccination. Mechanisms by which vaccines could induce ADEM include molecular mimicry and autoimmune activation, though these remain hypotheses (Figure 2).

This figure shows a step-by-step sequence of events from simultaneous vaccination to ADEM expression, with the hypothesized mechanism reflecting the findings from this case. The process begins with the administration of the influenza vaccine, which triggers activation of the immune system characterized by T- and B-cell responses. Molecular mimicry has been proposed as the key mechanism, and cross-reactivity occurs because of the structural similarity of the vaccine antigen to CNS myelin proteins. As a result, an autoimmune attack on CNS myelin occurs, causing demyelination. Neurological symptoms such as motor dysfunction and cognitive changes are consistent with the clinical picture of ADEM.

Importantly, vaccination remains a highly recommended preventive measure, given the much higher risk of complications, including ADEM, from natural influenza infections. Physicians should prioritize post-vaccination monitoring and ensure the prompt reporting of any adverse events to improve vaccine safety data [17,18]. Previous studies have suggested that repeated annual influenza vaccinations may increase the risk of developing ADEM in some children, raising concerns about cumulative immune activation [16,19].

Further research is needed to determine the exact mechanisms through which influenza vaccines might contribute to the development of ADEM. Large-scale epidemiological studies and detailed case reports will be essential to clarify the relationship between influenza vaccination and ADEM, as well as to identify potential risk factors for vaccine-associated CNS complications [16,17,18,19]. Understanding these mechanisms will be crucial not only for refining vaccine safety guidelines but also for developing strategies to mitigate risk in susceptible populations. Future studies should also investigate potential genetic or immunological predispositions that might render certain individuals more vulnerable to ADEM following vaccination [16,17,19]. This could lead to the development of personalized vaccination strategies, further optimizing the balance between vaccine efficacy and safety. Moreover, such research could also illuminate ways to improve vaccine composition, including adjuvants and formulation, to minimize adverse immune responses without compromising the protective effects of vaccines.

Given the benefits of influenza vaccination in preventing serious illness and the overall safety profile of influenza vaccines, it remains recommended that children receive annual vaccinations. However, healthcare providers should remain vigilant for any adverse events following vaccination and consider delaying or spacing out vaccinations when there is a history of previous adverse reactions [20,21,22,23,24].

## 4. Limitation of This Case Study

A notable limitation of this case study is the inability to definitively determine which of the two vaccines—the seasonal influenza vaccine or the H1N1 pandemic vaccine—was the cause of the acute disseminated encephalomyelitis (ADEM) in this patient. Both vaccines were administered simultaneously, and while both have been associated with ADEM in the literature, the exact mechanism by which they might contribute to the development of this rare complication remains unclear [20,21]. The lack of a controlled trial or cohort of similarly affected children who received only one of the vaccines makes it impossible to draw firm conclusions regarding the specific risk of each vaccine. Furthermore, while other factors such as genetic predisposition or a history of autoimmune disorders could have played a role, no definitive data exist to support these factors in this patient [25]. Additionally, the lack of a longitudinal follow-up limits our ability to assess the long-term impact of the vaccination on the patient’s health, and whether any subtle effects may appear over time. As such, further research, ideally through larger cohort studies or randomized controlled trials, is necessary to elucidate the individual contributions of seasonal and pandemic influenza vaccines to the risk of ADEM in pediatric populations [10,11,14].

Moreover, studies should aim to include diverse populations and explore additional environmental factors that may interact with vaccination to increase the likelihood of ADEM, thereby providing a more comprehensive understanding of this rare adverse event.

## 5. Conclusions

This case highlights the rare occurrence of ADEM following simultaneous seasonal and H1N1 influenza vaccination. While the exact cause remains unclear, it underscores the need for careful monitoring of adverse events, particularly in children. Despite the low risk of ADEM, the benefits of vaccination in preventing severe influenza complications outweigh the risks. Further research is needed to understand the mechanisms of vaccine-associated ADEM and improve safety guidelines.

## Data Availability

Data are contained within the article.
